# Treatment of Severe Comminutive Fractures of Long Bones

**Published:** 1919-01

**Authors:** 


***Treatment of Severe Comminutive Fractures of Long Bones: Complete Excision, Delayed Primary Suture, and Osteosynthesis or Grafting.*** By MM. R. Leriche and A. Policard. *Presse Médicale*, October 17, 1918.

The actual method of treating very severe comminutive bursting of long bones consists of the very careful removal of all small, loose fragments of bone and the very strict sparing of all fragments of a certain size (3 or 4 cm.) still attached to the muscles or the diaphyseal periosteum. The purpose is to cause as small a loss of bony substance as possible.

The authors do not agree with this conservative form of treatment.

(1) They consider that the necessity of sparing a few fairly attached fragments may hinder considerably the thorough cleansing of all recesses of the wound. A small amount of dead or devitalized tissue may very easily be left behind, and be responsible later on for some secondary infection.

The fear of detaching completely some very loosely attached sequestrum may cause to be left behind some minute splinters, and dust of bone. A fistula may then develop after the suturing of the wound and greatly interfere with the repairing process of osteogenesis, or of grafting.

(2) The authors insist upon the constant presence, in such wounds, of innumerable small foreign particles, invisible to the naked eye. They are enclosed within tissues which are quite sound in appearance along the whole track of the missile, and may be held responsible for many cases of pseudarthrosis.

They are well tolerated at first by the tissues, occasion no inflammation and do not hinder the success of primary suture; but fibrous cysts develop around them, and these may completely prevent the penetration of the newly developed bone.

(3) The control of the fragments is impossible and displacements with unavoidable muscular contractions prove very frequent. Thus instead of helping in the repairing process, these sequestra are of no use at all.

(4) The vitality of these splinters is generally reduced. If their connections with the living bone are not sufficient, and if there is any infection, they are gradually resorbed and a pseudarthroses develops.

The authors propose the following technic: early and complete esquillectomy in order to secure immediate asepsis of the wound; three days later delayed primary suture, in several layers, of the soft parts; osteosynthesis or grafting, 3 or 4 days after the removal of the threads, — that is, 15 to 20 days after suturing.

Osteosynthesis and bone grafts are both to be recommended in certain definite cases.

Osteosynthesis should be resorted to in the limbs with single bones, and grafting when there is loss of substance in a double bone, either in the forearm or in the leg. Grafting does not seem advisable for the humerus or femur unless there is really a considerable loss of substance.

The authors resorted to their new technic in 9 cases during one year. They performed 4 bone grafts and 5 osteosyntheses. The results proved excellent in all cases.

